# Stability of TPU/PP Blends Exposed to UV Radiation for Industrial Applications

**DOI:** 10.3390/polym17131842

**Published:** 2025-06-30

**Authors:** Carlos Vargas-Isaza, Jose A. Tamayo, Libia M. Baena, Juan Felipe Santa Marin, Adrian José Benitez-Lozano

**Affiliations:** 1 Grupo de Investigación Calidad, Metrología y Producción, Instituto Tecnológico Metropolitano, Medellin 050034, Colombia; carlosvargas@itm.edu.co (C.V.-I.); josetamayo@itm.edu.co (J.A.T.); libiabaena@itm.edu.co (L.M.B.); 2 Facultad de Minas, Universidad Nacional de Colombia, Medellin 050034, Colombia; jfsanta@unal.edu.co

**Keywords:** characterization methods, TPU, PP, FTIR analysis, TGA, SEM

## Abstract

Thermoplastic polyurethanes (TPUs) have found diverse applications across different industries, which expose the material to various environmental conditions. Among these, UV radiation stands out as one of the most aggressive, leading to significant degradation in polymers. Considering this, this study explores the use of commercial additives, such as polyethylene masterbatches (MB), and their effectiveness as inhibitors of UV radiation-induced degradation. In addition, it investigates the possibility of blending high-performance polymers, such as TPU, with commodity polymers, such as polypropylenes. The prepared blends were evaluated via thermogravimetric analysis, infrared and electron microscopy, hardness and tensile strength assessments, and scanning electron microscopy before and after 320 h of exposure to accelerated aging. The findings suggest that the adequate incorporation of additives in blends can help to reduce the harmful effects caused by UV radiation on the polymeric materials.

## 1. Introduction

Thermoplastic polyurethanes (TPUs) first appeared on the market in the 1960s as linear block copolymers with alternating hard and soft segments. The soft segments (SSs) are formed by large and flexible chains of diols, while the hard segments (HSs) are commonly formed by alternating isocyanates and short-chain extension sequences. The chemical incompatibility between SSs and HSs generates an isolated two-phase system with small hard domains at the nanometric scale. The HSs, particularly, play a crucial role in bonding polymer chains together and preventing them from flowing at the working temperature. In addition, upon reaching the softening temperature of the hard phase, the polymer flows and can be easily and reversibly shaped. Thanks to this chemical configuration, TPUs are known for their versatility in terms of formulation and ease of processing [1,2,3].

Some common applications of TPU include photovoltaic cell encapsulation and the manufacturing of interior parts for automobiles, footwear, flexible hoses and tubes, sports equipment, textiles, implantable medical devices, mine equipment, and cattle identification tags [2]. Although some of these applications involve outdoor use, few authors have evaluated the effect of ultraviolet (UV) radiation on this type of polymer. For instance, Palak et al. [4] assessed the UV protection of TPU electrospun with nano clays and titanium dioxide and obtained promising results. Regarding blends manufactured with TPU and polyolefins, such as polypropylene (PP), various studies have examined their performance in terms of mechanical properties [5,6,7], thermal characteristics [6,8,9], and blend compatibilization [9,10,11,12], among other aspects [13,14,15]. However, the effect of UV radiation on the final properties of these blends has not yet been explored.

Blends incorporating TPU have found several applications in recent years, with a large number of them focused on improving a material’s mechanical properties. For example, to enhance mechanical stability, a specific amount of TPU was added to polyethersulfone (PES) in a recent study [16]. Two different materials were separately incorporated into the membrane matrix: tannic acid-polyethylene glycol (TA-PEG) and aminated metal–organic framework-101 (Fe) (MIL-101(Fe)). Subsequently, each component was integrated into the membrane matrix in an equal mass ratio, and the outcomes were compared [17]. In recent decades, there has also been significant progress in the preparation and design of nanofiltration (NF) membranes for use in dye rejection applications across a variety of industries, including the textile sector. In a study, NF membranes were fabricated by incorporating varying amounts of PES, TPU, and APTS functionalized-graphene oxide (GO-APTS) nanosheets [18]. The improved Hummer’s method for producing graphene oxide (GO) was used to prepare the GO-APTS nanosheets, to which (3-aminopropyl) triethoxysilane (APTS) was added [16].

In a similar vein, regarding studies on TPU, the authors of [17] prepared membranes by blending PES with TPU to enhance the mechanical properties of PES membranes. To prevent macro-void formation and improve mechanical stability, they fabricated these membranes by employing the vapor-induced phase separation coupled with a non-solvent-induced phase separation (VIPS-NIPS) method [17]. Likewise, a novel manufacturing technique known as long-fiber-reinforced thermoplastic (LFT) was introduced in [19] to create continuous flexible composite carbon fibers and fabrics. To produce TPU blends with the potential to enhance interfacial bonding, a polyester hot-melt adhesive (MPTU) was added to TPU. Subsequently, the TPU blends were extruded to enwrap carbon fibers and form an outer layer. The effects of the TPU blends on the composite were then examined to identify the optimal parameters. According to the findings, at a TPU/MPTU ratio of 85/15 wt.%, the blends exhibited good miscibility, and the fiber tows showed optimal clustering and knot properties [18].

In [20], ternary nanocomposites made up of carboxylated carbon nanotubes (CNTs) dispersed in a mixture of two immiscible polymers, namely poly(L, lactide) (PLLA) and TPU, were characterized using transmission electron microscopy, temperature-modulated differential scanning calorimetry, and broadband dielectric spectroscopy. These nanocomposite blends were obtained through the melt compounding of PLLA and TPU, incorporating 0.2 wt.% CNT. This process was carried out both with and without a Joncryl^®^ ADR chain extender for PLLA, resulting in the creation of reactive and non-reactive melt-mixed samples [20].

As mentioned previously, some studies have focused on improving the mechanical properties of TPU. Particularly, in [19], TPU was modified through a blending method using polyether-block-amide elastomer (PEBA). Since the two materials were not very compatible, a polyurethane known as E-PU-I, with one end blocked with an isocyanate group and the other end blocked with two epoxy groups, was created as a compatibilizer. Additionally, using a torque rheometer, several TPU/PEBA blends with various components were prepared, and the impacts of E-PU-I on the blends’ microstructure, phase−interface interaction, rheological characteristics, and mechanical properties were investigated [19].

Despite the benefits provided by nanomanufacturing, in nanocellular foam production, the difficulties lie in achieving cell sizes below 100 nm with low relative densities. In a referenced study [21], three TPUs with varying hardnesses were combined with poly (methyl methacrylate) (PMMA) to examine the effects of TPUs on foam density and the nanocellular structure. According to the findings, the blends’ nanostructure was mostly controlled by the viscosity of TPU. Moreover, a well-dispersed system with the smallest TPU particle size (less than 100 nm) was produced when 2 wt.% TPU was blended with PMMA [21].

In recent biomedical applications, esophageal stents have been widely used for the palliative therapy of esophageal cancer in clinical settings. However, commercial esophageal stents are usually not customized according to the patient’s esophageal geometry but rather are manufactured in predetermined sizes. In [22], a TPU/Poly-ε-caprolactone (PCL) blended esophageal stent was created by combining a melt-blending method with a 3D printing technique.

There are many studies regarding PP and TPU blend benefits, and one of them focuses on the impact-resistant polypropylene/thermoplastic polyurethane blends with compatible effects of maleic anhydride on thermal degradation properties and crystallization behaviors [23,24,25,26]. Other studies focus on recycling thermoplastic materials to attain the purposes of recycling, reuse, and sustainability. Polypropylene (PP) and toughening thermoplastic polyurethane (TTPU) are melt blended for two cycles, and a small amount of compatibilizer (i.e., polypropylene grafted maleic anhydride, PP-g-MA) is added during the process. The impact test results have proven that the incorporation of 20 wt.% of TTPU and 5 wt.% of compatibilizer helps to improve the inter-phase problem, thereby yielding an impact strength of 63.01 J/g. The tensile strength test results show that the presence of PP compensates for the insufficient rigidity and high production cost of TTPU [27]. In other studies, polypropylene (PP) and thermoplastic polyurethane (TTPU) are blended, resulting in remarkable blends in the field of recyclable polymers, and both materials (PP and TPU) can be properly heated to reform and be reused due to the inherent characteristics of thermoplastic materials. The utilization of excess plastic waste through an efficient and convenient melt extrusion process is increasingly recognized as an eco-friendly strategy aligned with sustainable development goals [9]. In the context of thermoplastic polyurethane (TPU), the incorporation of stabilizers during melt processing not only enhances the thermal and UV stability of the material but also facilitates the recycling and repurposing of polymeric waste. This approach contributes to a circular materials economy by extending the service life of TPU-based products and reducing environmental impact, thereby supporting green innovation and responsible material management on our planet.

Regarding PE and polyurethane blends, one benefit of paint adhesion is that polyolefins have low free surface energy that prevents good wettability of adhesives or paint emulsions to their surface [28].

When exposed to outdoor environments, most polymers are highly susceptible to degradation, with UV radiation between 280 and 400 nm being one of the environmental factors affecting polymer stability. This degradation occurs because the energy of the UV photons from sunlight is comparable to the dissociation energies of the covalent bonds in the polymer, which causes a chemical reaction promoting the breaking of covalent bonds in carbonate chains and the generation of free radicals. As a result, the polymer loses molecular weight, and the impact of such ruptures within the polymeric chains is evident in optical properties (such as color and gloss loss), diminished mechanical properties, and the formation of microcracks or material fractures [2,3,23].

In this study, we evaluated four blends to determine if the additives used can improve UV protection, thereby preserving the physical and chemical properties of the polymeric materials obtained after 320 h of exposure to UV radiation in an accelerated aging chamber. Additionally, we present characterization techniques focused on the aforementioned mixtures and test tube products, which were subjected to highly invasive and aggressive atmospheres. The findings are applicable in technological and industrial settings, marking an innovative contribution to the field. This work presents characterization techniques focused on the mixtures mentioned and test tube products, subjected to highly invasive and aggressive atmospheres, usable in technological and industrial applications, as an innovative research work in the field.

## 2. Materials and Methods

### 2.1. Materials

The TPU employed in this study was provided by Bayer A.G. (Leverkusen, Germany) under the Desmopan 3491A trademark (Ester grade, density = 1.2 g/cm^3^, Shore A hardness = 92). The polypropylene homopolymer we used was Moplen PH 1310, a commercial product with a melt flow index of 13 (g/10 min) at 230 °C, sourced from Petroquim (Hualpén, Chile). Additionally, a commercial PE masterbatch (MB) with UV stabilizers was obtained from Arcolor (Itagui, Colombia) to offer UV protection to the blends at a percentage of 3–5%, as recommended by the manufacturer.

### 2.2. Blend Preparation and Selection

The blends were prepared through melt blending using a THERMO torque rheometer (POLYLAB QC) (THERMO, Bacchus Marsh, Australia) operating at 190 °C and 60 rpm for 15 min. Initially, the TPU was introduced into the rheometer chamber for the first three minutes. Once the torque stabilized, PE masterbatch (MB) and PP were added to the melted TPU. Subsequently, the blends were compression molded at 190 °C for 7 min using a constant pressure (10 MPa) and cooled to room temperature to form sheets. After compression molding, the sheets were cut into samples for accelerated aging tests and chemical and mechanical characterization.

**Figure 1:**
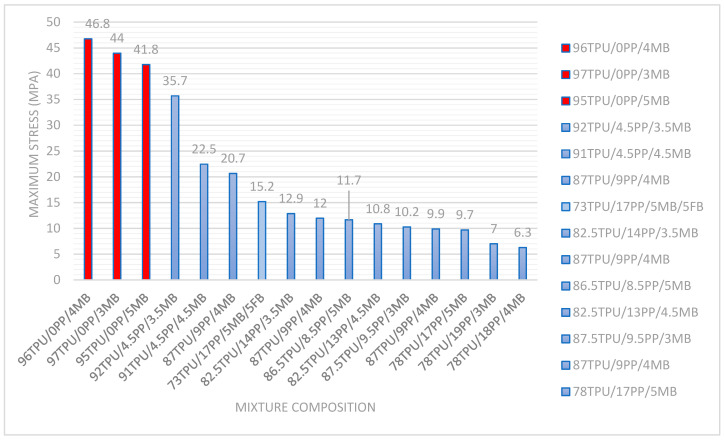
Results of the preliminary tests and composition of the evaluated blends. Maximum stress vs. composition.

The initial assessment involved evaluating the maximum resistance of various mixtures, as illustrated in Figure 1. The figure also shows the chemical composition (TPU, PP, and MB) of each sample. After these initial tests, it was concluded that TPU contributed to the mechanical resistance and that the concentration of TPU must be higher than 90% wt.% in each formulation. Following preliminary testing, four blends were selected for evaluation in accelerated UV tests, as indicated in Table 1.

The initial assessment involved evaluating the maximum resistance in tensile tests of various mixtures according to ASTM D638 [29], as illustrated in Figure 1. According to standard ASTM D638 [29], for statistical reliability reasons, the test must include at least five specimens for each sample in the case of isotropic materials. For each series of tests, the arithmetic mean was calculated of all values obtained and was reported as the “average value” for the particular property (in this case, maximum tensile strength). Figure 1 and images in Section 3.4 were constructed according to this standard method.

The same figure also shows the chemical composition (TPU, PP, and MB) of each sample. The first number indicates the amount of TPU in weight percentage. The second number is the amount of PP and the last number indicates the amount of PE masterbatch UV. After these initial tests, it was concluded that TPU contributed to mechanical resistance and that the concentration of TPU must be higher than 90% wt.% in each formulation. Following preliminary testing, four blends were selected for evaluation in accelerated UV tests, as indicated in Table 1.

Regarding PE being used as a masterbatch, one reason for its selection in the blend composition is that it is an economical and commercially available material. But one of the main reasons for selecting PE as a masterbatch is that, if MB has to be formulated based on TPU or other more compatible matrices, it would probably be more complicated and, of course, more expensive, as it is customized. On the other hand, since it is flexible, it could couple with the mismatch in deformation that might be caused by using other polymers. According to the manufacturing company (Arcolor), the masterbatch (PE) used has some properties that improve UV light performance, and, in the material datasheet, the light performance is excellent (8 on the scale between 1 and 8 for light fastness) [30].

As the aim of this study is to explore the feasibility of acquiring TPU/PP blends without coupling agents while integrating a UV additive and seeking to diminish the TPU quantity by incorporating a more cost-effective material, such as PP, Table 1 provides four material and blend options. Based on previous results reported in Figure 1, it was decided to select blends with the highest PP content without significantly compromising the tensile strength compared to TPU blends only with the additive. Following this criterion, Blend 1 (91TPU/4.5 MB/4.5 PP) and Blend 3 (90TPU/4 MB/6 PP) were chosen. The latter, although not evaluated in the study reported in Figure 1, proposes a composition with a maximum content of 6% PP, as previous blends evaluated with contents between 9 and 19% PP showed very low tensile strength performances. Blend 2 (97TPU/3 MB) was evaluated to observe the effect of the UV additive for subsequent tests compared to TPU without additives and without PP (“Blend 4”). The contents of UV additives in TPU/PP blends were determined according to the supplier’s recommendations, ranging from 3 to 5%, aiming to enhance the UV protection of the blends.

Some authors have used mixtures of antioxidants (AO) and Hindered Amine Light Stabilizers (HALS) to provide UV protection for TPU [31,32,33]. Other authors have used mixtures of antioxidants, composite additives (containing ZnO, CeO_2_), and HALS to protect PU films [19]. In this work, the authors used a commercial product to provide UV protection to the blends. According to the manufacturer, the product is a mixture of organic pigments and/or inorganic additives in a linear polyethylene matrix. Since the manufacturer did not provide additional information about the composition of the commercial product, the authors decided to perform some tests (energy-dispersive spectrometry coupled with the scanning-electron microscope and infrared analysis) to conclude that the commercial product is a mixture of quenchers, HALS and composite additives.

### 2.3. Accelerated Aging via UV Radiation

The blends underwent degradation after exposure to UV radiation for 320 h in a QUV-accelerated aging chamber from Q-LAB (Westlake, OH, USA). This chamber was equipped with four fluorescence lamps (UVA-340) on each side, and the exposure followed the established parameters in cycle 1 based on the ASTM G154 standard [34]. The samples were subjected to eight hours of UV radiation at an intensity of 0.89 +/− 0.1 W/m^2^ and a temperature of 60 °C, followed by four hours of condensation at 50 °C.

### 2.4. Chemical and Mechanical Characterization

In this study, the degradation of blends after exposure to UV radiation was evaluated. In addition, their chemical and mechanical properties before and after 320 h of exposure in a UV radiation chamber were analyzed.

The thermal decomposition and maximum temperature of degradation of the blends were assessed by thermogravimetric analysis (TGA) using a simultaneous TGA/DSC analyzer from TA Instruments from New Castle, DE, USA (SDTQ600 model). The heating rate was set at 10 °C/min, reaching 600 °C in a nitrogen atmosphere, and then air was used up to 900 °C at the same heating rate. Changes in chemical structure due to reaction with UV radiation were examined via Fourier transform infrared spectroscopy with attenuated total reflection (FTIR-ATR) on a Shimadzu Tracer IR-100 equipment (Shimadzu, Kyoto, Japan) in the range of 4000 cm^−1^ to 500 cm^−1^. A total of 16 scans and a resolution of 1 cm^−1^ were used for the tests. Morphological changes resulting from degradation were analyzed by scanning electron microscopy (SEM) (JEOL JSM 6490 LV, JEOL, Tokyo, Japan) coupled with energy dispersive X-ray spectroscopy (EDS) (OXFORD INCA Penta FET-x3, Oxford Instruments, Abingdon, UK). Prior to analysis, the samples were coated with a thin gold layer using a sputtering system.

The effect of UV radiation-induced degradation on the mechanical properties of the samples was determined by hardness measurements using Shore A and Shore D hardness testers (Bareiss brand, Oberdischingen, Germany) following the DIN 53505-ISO 868 ISO 48-4:2018 [35] standards, respectively. Tensile strength was tested in a universal machine (Shimadzu AG-100 kNX) equipped with a Shimadzu SPL-10kNA load cell.

#### Surface Degradation by Determining Color Degradation

The authors also evaluated the surface degradation by determining the color degradation. High-resolution images of the samples before and after UV degradation were taken using a controlled light source. The images were taken at the zones with the color variation after the UV tests. After that, the image’s histograms were analyzed using digital image processing software. The changes in the RGB histograms, mean intensity, and energy were compared, and the variation was also quantified.

## 3. Results and Discussion

### 3.1. Thermogravimetric Analysis (TGA)

**Figure 2:**
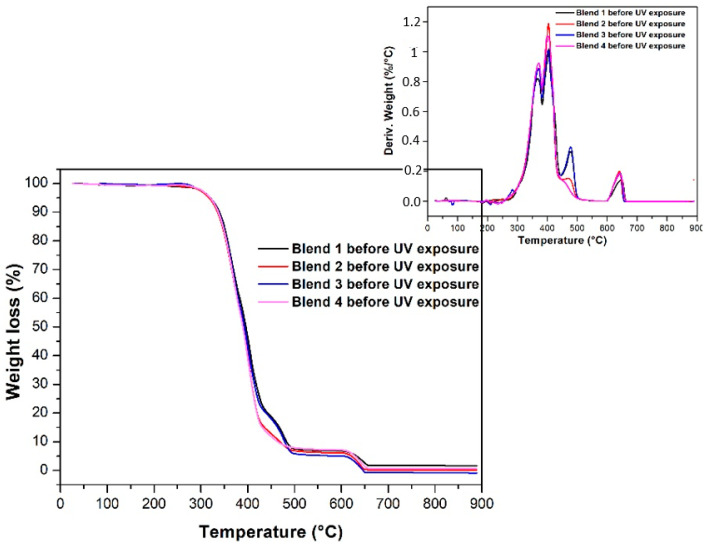
Thermogravimetric curves of Blends 1 to 4 before being exposed to accelerated aging in a UV radiation chamber.

Figure 2 shows the thermogravimetric curves obtained for Blends 1 to 4 before being exposed to accelerated aging in a UV radiation chamber. As observed, the TPU blends decomposed in two stages at temperatures between 280 °C and 450 °C, with two peaks at 370 °C and 400 °C. The first stage of TPU’s thermal degradation is associated with the breakdown of the urethane bonds in the hard segments, while the second stage is linked to the fragmentation of the polyol groups in the soft segments of the polymer [28,29]. Notably, the thermogravimetric curves of Blends 1 and 3 exhibited a displacement between 400 °C and 500 °C, attributed to the PP content in both samples. This is because the thermal degradation of pure PP typically occurs between 390 °C and 490 °C [30].

**Figure 3:**
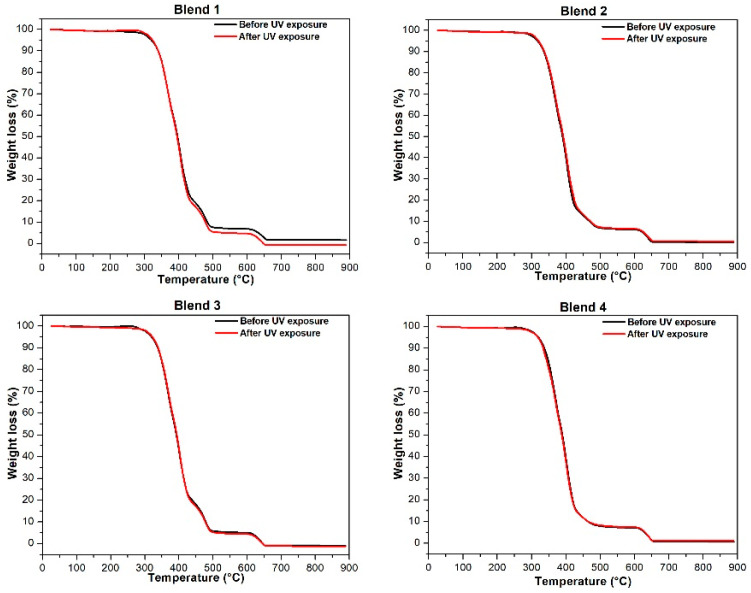
Thermogravimetric curves of all blends before and after 320 h of exposure in a UV radiation chamber.

**Figure 4:**
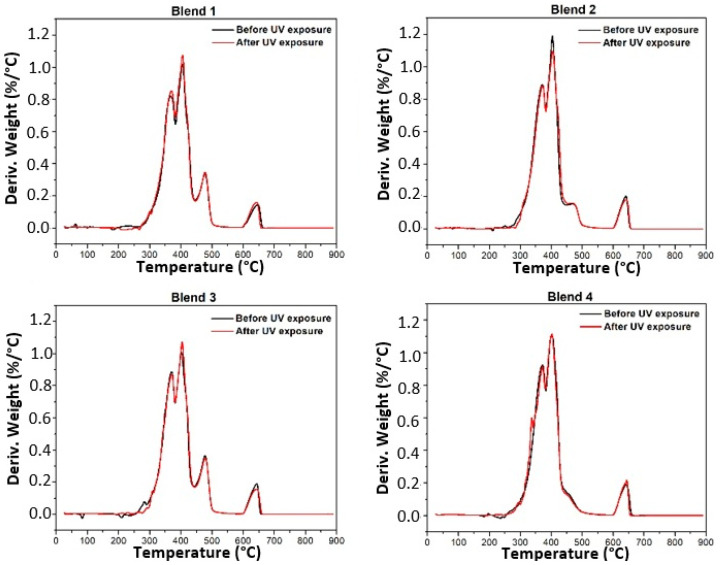
Derivative thermogravimetric curves of all blends before and after 320 h of exposure in a UV radiation chamber.

Figure 3 and Figure 4 depict the thermogravimetric curves and respective derivative curves obtained for Blends 1 to 4 before and after 320 h of exposure in a UV radiation chamber. Interestingly, no changes are observed in the curves for any of the blends post-UV radiation. The samples not exposed to UV radiation show no displacement in the curves towards lower degradation temperatures; instead, the curves perfectly overlap.

Table 2 reports the maximum temperature of degradation of each blend, revealing no significant changes for all blends subjected to accelerated aging. These results conclusively demonstrate that, following 320 h of exposure to UV radiation, none of the evaluated blends experienced changes in thermal stability, and their thermal behavior remained constant.

### 3.2. Fourier Transform Infrared (FTIR) Analysis

**Figure 5:**
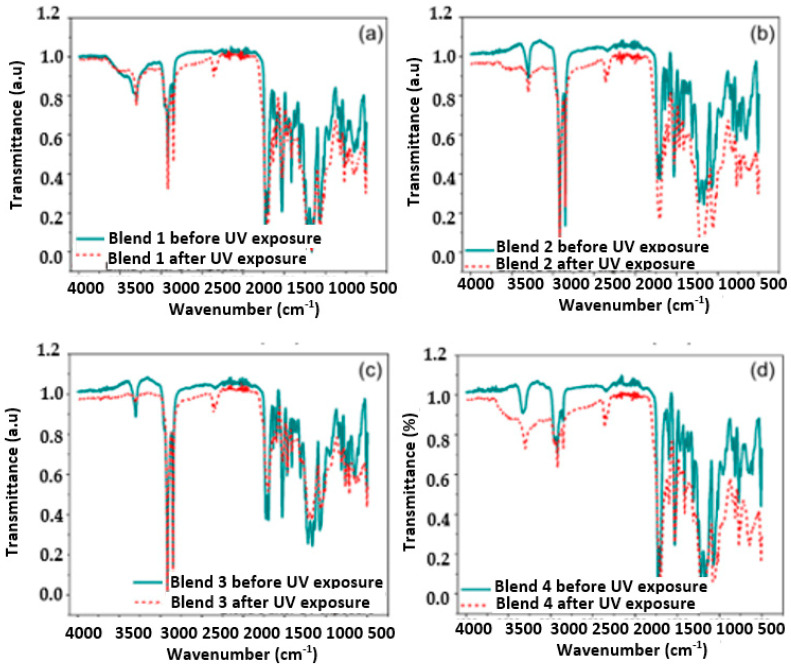
Infrared spectra of the analyzed samples before and after 320 h of exposure in a UV radiation chamber: (**a**) Blend 1, (**b**) Blend 2, (**c**) Blend 3, (**d**) Blend 4.

Chemical changes in the blends under analysis were evaluated by FTIR spectroscopy both before and after exposure to 320 h of UV radiation in the chamber. Figure 5 presents the IR spectra obtained for the blends. Notably, the characteristic spectrum of the TPU polymer was observed in all the blends, featuring a double peak band of high intensity associated with the PE masterbatch and the polypropylene (located between 2700 cm^−1^ and 3000 cm^−1^ for Blends 1 to 3 [36,37,38]).

**Figure 6:**
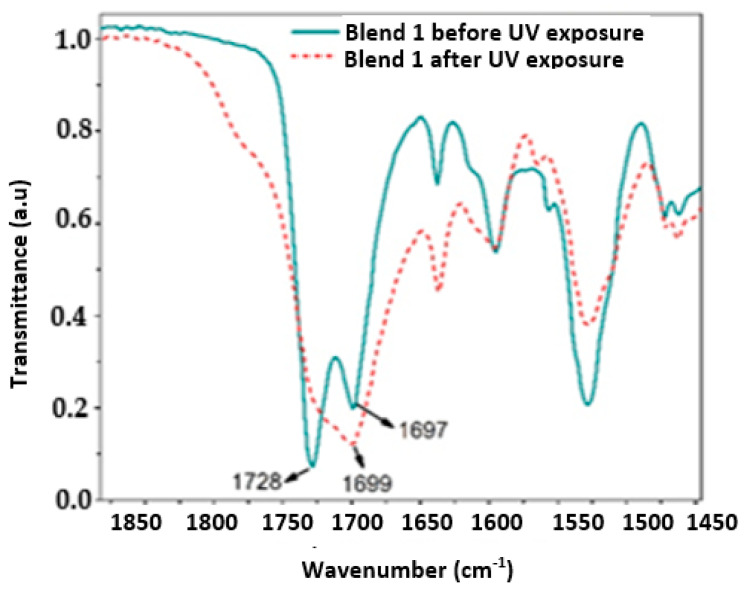
Close up of the IR spectrum in the region between 1900 cm^−1^ and 1450 cm^−1^ for the evaluated samples of Blend 1.

**Figure 7:**
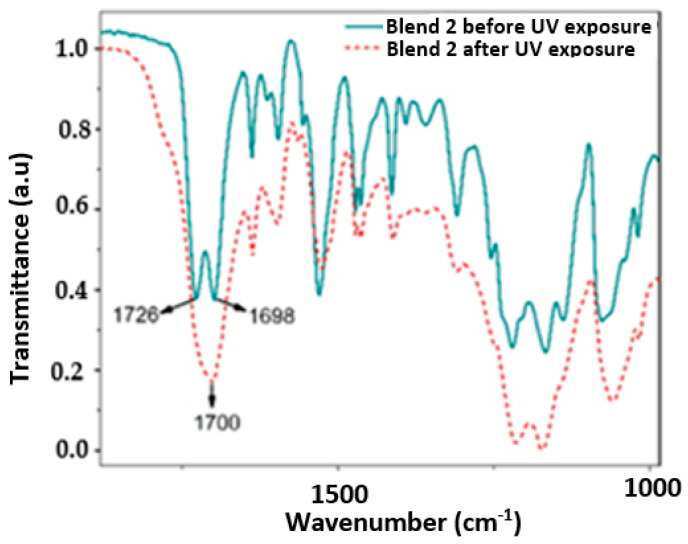
Close up of the IR spectrum in the region between 1800 cm^−1^ and 1000 cm^−1^ for the evaluated samples of Blend 2.

**Figure 8:**
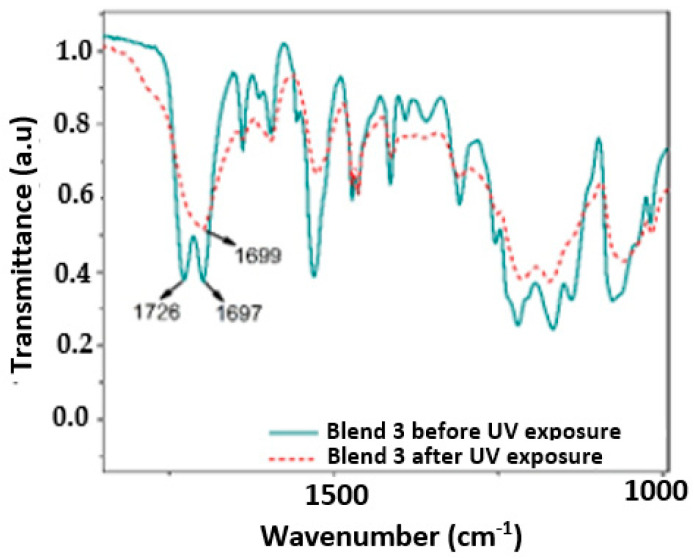
Close up of the IR spectrum in the region between 1800 cm^−1^ and 1000 cm^−1^ for the evaluated samples of Blend 3.

**Figure 9:**
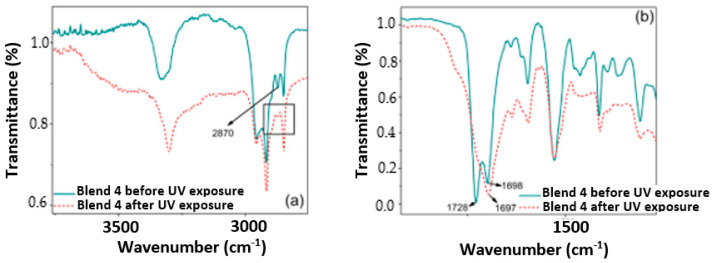
Close up of the IR spectrum for the evaluated samples of Blend 4: (**a**) in the region from 3500 cm^−1^ to 2750 cm^−1^ and (**b**) in the region from 1800 cm^−1^ to 1000 cm^−1^.

Figure 6, Figure 7, Figure 8 and Figure 9 show a close up of the spectrum region, offering a detailed view of the double peak band located between 1750 cm^−1^ and 1690 cm^−1^. The peak signal at 1730 cm^−1^ and another around 1700 cm^−1^ correspond to stretching vibrations of the carbonyl groups (C=O), ester, and urethane, respectively. Following 320 h of exposure in a UV radiation chamber, this doublet disappeared in all blends, and a signal was formed around 1700 cm^−1^. This change in the spectrum indicates a degradation of the ester and urethane functional groups, along with the formation of new carbonyl groups. In Blends 1, 2, and 3, a slight decrease in signal intensity was observed around 1530 cm^−1^. This signal is associated with the deformation band of the N-H bonds and the stretching vibration band of the C-N bonds in the urethane groups [3,39,40,41].

The band around 3330 cm^−1^ corresponds to the stretching vibration of the H bond of the N-H group in the urethane. As shown in Figure 5a, in the spectrum of the sample exposed to UV radiation, this band widened between 3050 cm^−1^ and 3650 cm^−1^. Blend 2, for its part, exhibited a widening of the band at 3300 cm^−1^, ranging from 3600 cm^−1^ to 3090 cm^−1^, although it was slightly lighter than that observed in Blend 4. This broadening may be linked to the formation of hydroxyl groups (-OH) induced by UV radiation, although stretching vibrations of primary amines were observed in the region between 3350 cm^−1^ and 3250 cm^−1^ [42,43]. Additionally, Blend 4 displayed a subtle change in the band between 3000 cm^−1^ and 2800 cm^−1^, with a significant reduction in the intensity of the peak located at 2870 cm^−1^. This band is associated with the stretching vibrations of the CH_2_ groups, and the decrease in signal intensity can be attributed to the oxidation of the polymeric chain [3,40,44].

Similar to how the decarboxylation of urethane groups can lead to the formation of primary amines, the formation of carbonyl and hydroxyl groups may suggest the formation of carboxyl groups induced by UV radiation [40]. The spectra of Blends 2 and 4 exhibited changes due to chemical oxidation, not only in the region from 1730 cm^−1^ to 1700 cm^−1^ but also in the 3330 cm^−1^ band. In addition, Blend 4 presented changes in the region between 3000 cm^−1^ and 2800 cm^−1^, indicating a possible degradation of the carbon chain. Importantly, Blends 2 and 4 contained the highest percentage of TPU by weight, especially Blend 4, with 100% TPU, which may indicate that the PP and polyethylene masterbatch added to Blends 1 and 3 inhibited, although not completely, the oxidative degradation of both blends.

### 3.3. Scanning Electron Microscopy (SEM) Analysis

**Figure 10:**
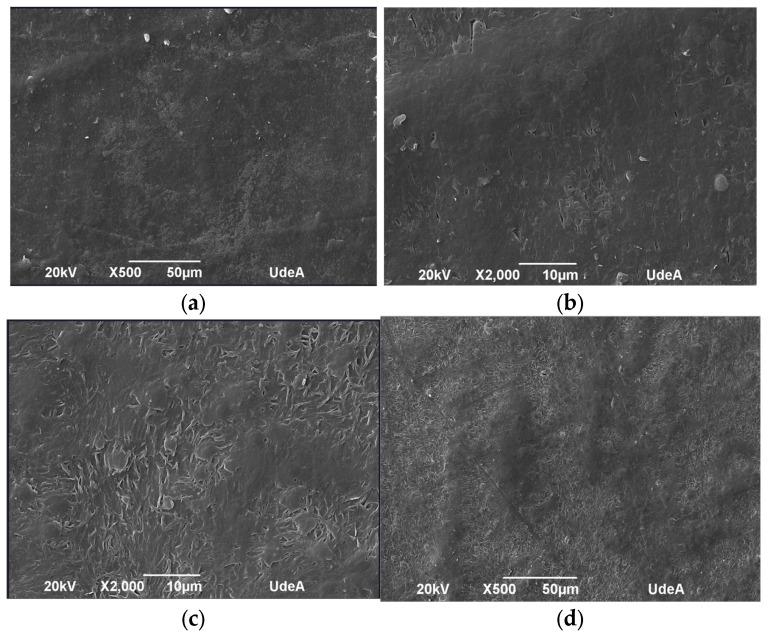
SEM micrographs of Blend 1 (**a**) before UV exposure (500×), (**b**) before UV exposure (2000×), (**c**) after 320 h of UV exposure (2000×), and (**d**) after 320 h of UV exposure (500×).

**Figure 11:**
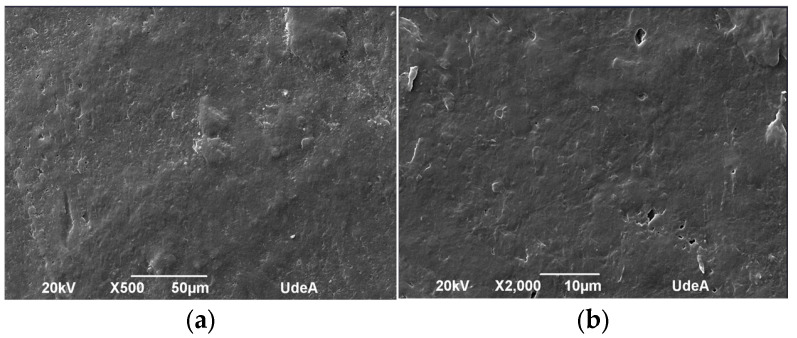

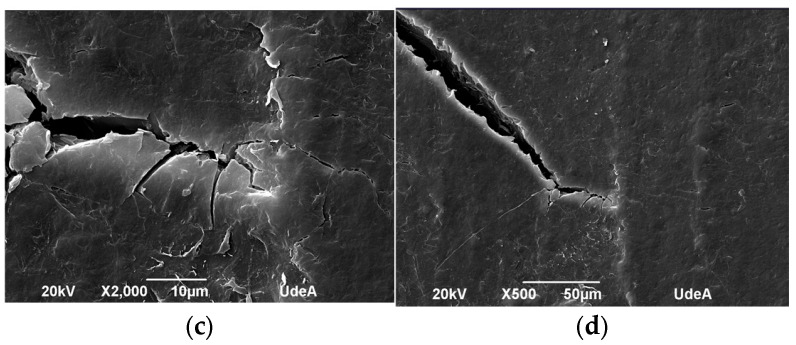
SEM micrographs of Blend 2 (**a**) before UV exposure (500×), (**b**) before UV exposure (2000×), (**c**) after 320 h of UV exposure (2000×), and (**d**) after 320 h of UV exposure (500×).

**Figure 12:**
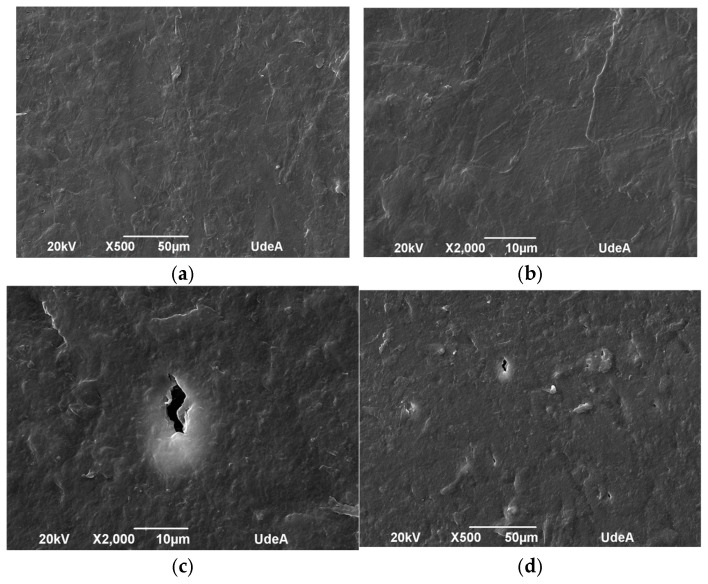
SEM micrographs of Blend 3 (**a**) before UV exposure (500×), (**b**) before UV exposure (2000×), (**c**) after 320 h of UV exposure (2000×), and (**d**) after 320 h of UV exposure (500×).

**Figure 13:**
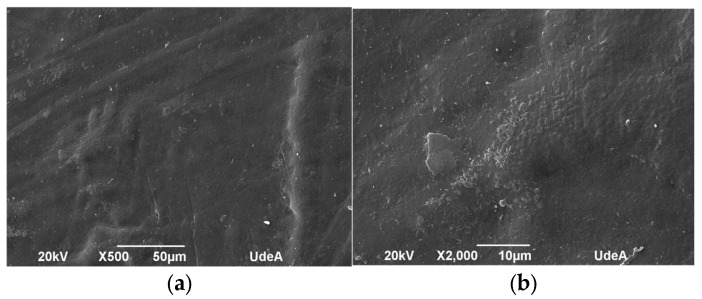

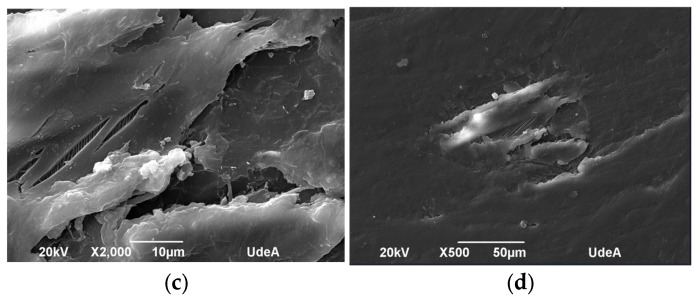
SEM micrographs of Blend 4 (**a**) before UV exposure (500×), (**b**) before UV exposure (2000×), (**c**) after 320 h of UV exposure (2000×), and (**d**) after 320 h of UV exposure (500×).

The microstructure of the samples was evaluated before and after UV aging to identify morphological changes induced by UV exposure. Figure 10, Figure 11, Figure 12 and Figure 13 depict the SEM micrographs of Blends 1, 2, 3, and 4 captured before and after 320 h of exposure in a UV radiation chamber. As observed, exposure to UV radiation caused morphological changes in the evaluated samples. When comparing the sample with exposure to the sample without exposure for Blend 1, we observed tears, cracks, and a disruption in surface homogeneity in the former, as illustrated in Figure 10c.

Regarding Blend 2, the SEM micrographs in Figure 11c,d revealed a considerable fracture and some surface cracks. While this blend exhibited some porosity before being exposed to UV radiation, the observed surface cracks do not seem to have originated from the existing pores before aging. Blend 3, for its part, as shown in Figure 12a,b, displayed a homogeneous surface with no pores; however, after exposure to UV radiation, surface cracks became noticeable. Finally, Blend 4 exhibited delamination and surface cracking following exposure to UV radiation. In general, after being exposed to accelerated aging in a UV radiation chamber, all the evaluated samples showed signs of physical degradation. Blends 2 and 4 exhibited noticeable physical defects at the surface. While the degradation of Blends 1, 3, and 4 was predominantly superficial, Blend 2 presented deeper cracks, indicating more deterioration and a higher degree of material embrittlement.

### 3.4. Analysis of Mechanical Properties

#### 3.4.1. Hardness

**Figure 14:**
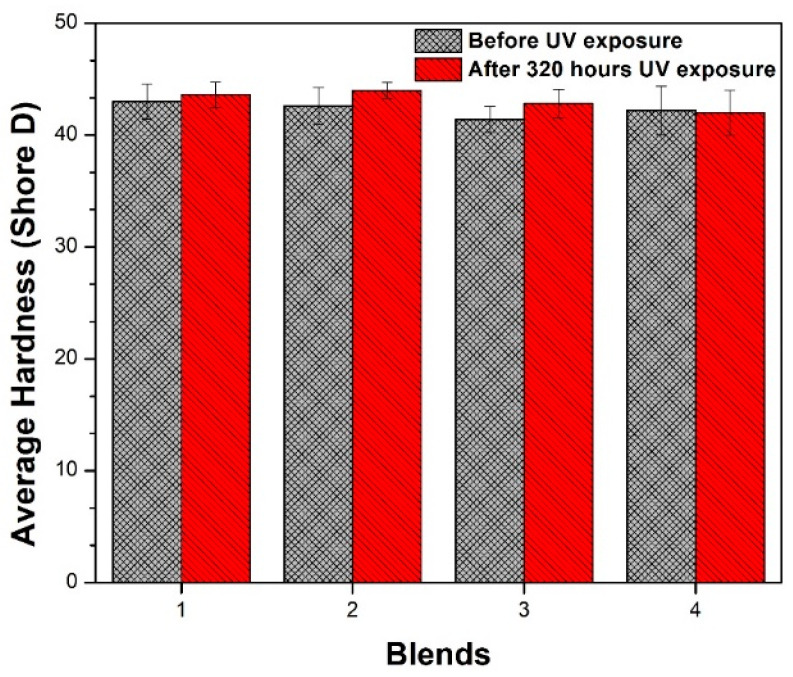
Average Shore D hardness of the four blends before and after 320 h of exposure in a UV radiation chamber.

For polymeric materials with Shore A hardness equal to or greater than 90, it is recommended to assess hardness using a Shore D tester [35,45]. In this study, five measurements of Shore A hardness were performed, and since the obtained values were close to 90, we opted to conduct the measurements using the Shore D hardness scale. The results, presented in Figure 14, depict the variation in Shore D values for the evaluated blends before and after 320 h of exposure in a UV radiation chamber. Importantly, five measurements were performed per sample to obtain an average and standard deviation. As can be seen in Figure 14, the average hardness of the blends slightly increased after exposure to UV radiation, except for Blend 4, which exhibited no significant change in hardness following exposure. The increments in hardness due to UV radiation were 1.4%, 3.3%, 3.4%, and 0.5% for the four blends. All increments are within the error of either the instrument or the experiment, as is shown in each deviation bar for each blend in Figure 14.

Exposure to UV radiation in the presence of oxygen triggers a photo-oxidation reaction in polymers, resulting in alterations to the chemical and macromolecular structure, which is manifested in chain scissions, cross-linking, or recrystallization. UV-induced aging causes the material to become harder because of an increase in macroscopic packing, which, in turn, elevates the density of the polymer due to modifications in amorphous regions. Chain breaking in these regions increases crystallinity, reducing the plastic deformation capacity and, consequently, increasing the hardness of the polymeric material [42,43,46,47]. In all the evaluated blends, a hardening phenomenon was observed, except for Blend 4, which lacked PP additives and PE masterbatch. This suggests that the additive content in the hardened TPU blends was not sufficient to reduce sensitivity to the photo-oxidative process for the materials obtained with Blends 1, 2, and 3.

#### 3.4.2. Tensile Strength

**Figure 15:**
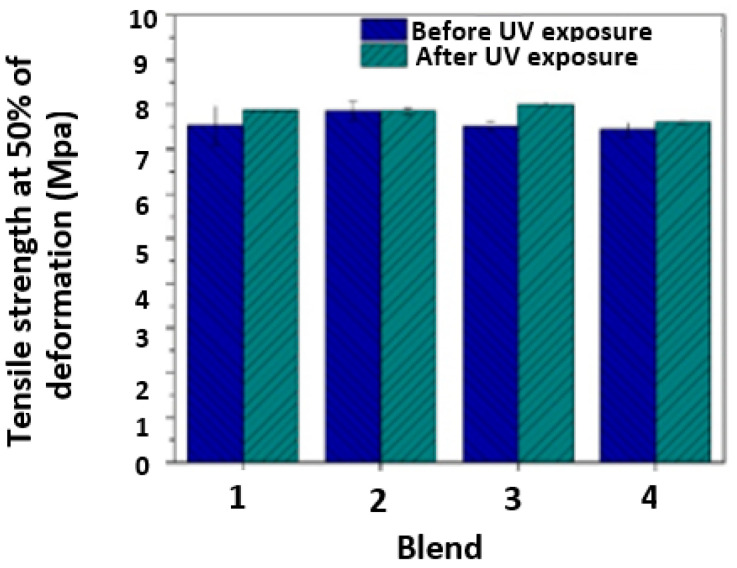
Average tensile strength at 50% strain of the four blends before and after 320 h of exposure in a UV radiation chamber.

**Figure 16:**
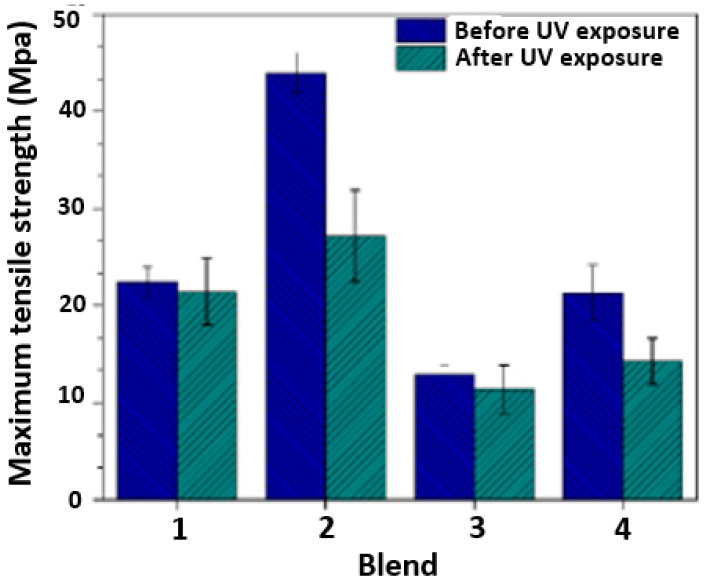
Maximum tensile strength of the four blends before and after 320 h of exposure in a UV radiation chamber.

Figure 15 shows the tensile strength at 50% strain of the four evaluated blends both before and after 320 h of exposure in a UV radiation chamber. At this strain level, we observed no noticeable negative impact of UV exposure on the tensile strength of the blends. However, there was a notable effect of UV exposure on maximum tensile strength. Specifically, there was a significant reduction in maximum tensile strength for Blends 2 and 4 (38% and 33%, respectively), as depicted in Figure 16. Conversely, for Blends 1 and 3, there was no significant reduction in maximum tensile strength for the samples evaluated after exposure to UV radiation.

According to these results, adding 4 wt.% of the PE masterbatch reduces the impact of UV exposure, and the reduction in tensile strength is not as severe. Additionally, maintaining a composition between 4 wt.% and 4.5 wt.% of PE masterbatch in the blend allows for the incorporation of PP without a significant reduction in maximum tensile strength.

Blend 2 (97TPU/3 MB) was evaluated, as shown in Figure 16, presenting maximum tensile strength before and after UV exposure compared to the rest. PE used as a masterbatch potentially increases the property; because this MB is flexible, it could couple with the mismatch in deformation that might be caused by using other polymers. This behavior could be incremented with these mechanical properties in comparison with the other blends evaluated. The use of this PE as a masterbatch allows for the development of improved polymeric compounds in regard to mechanical, thermal, and barrier properties, among others.

#### 3.4.3. Surface Degradation Color Analysis Results

**Figure 17:**
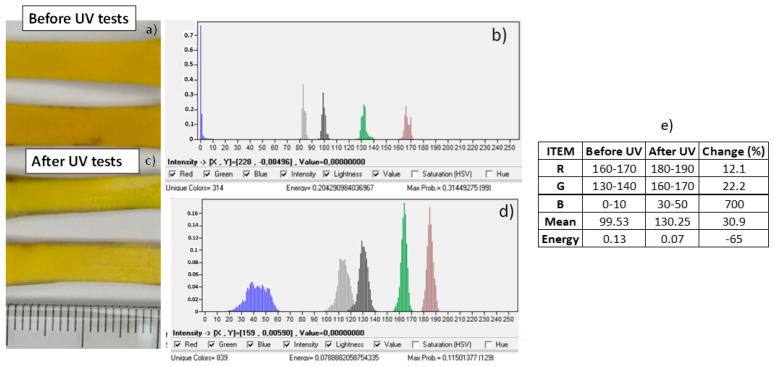
(**a**–**e**) Results of color analysis of samples before and after UV tests.

Figure 17 shows the surfaces of the samples before and after UV degradation. The color change after UV tests can be observed. The color of the samples after UV degradation (see Figure 17a,b) is paler. The histogram (see Figure 17c,d and table in Figure 17e) shows that the RGB histograms were modified toward high intensities (whiter resultant color). The most significant change is found with the blue histogram, with a change of 700%. This change in blue color is expected, since blue chromophores in dyes are quickly degraded because they absorb UV sunlight. The energy for the samples also decreases and the mean intensity increases. Ultraviolet rays cause a fading of the color of polymers because they can break down chemical bonds and create oxygen radicals. Specifically, the bleach effect for polyurethane has been reported by other authors [40], as the IR change and yellowing of polyurethane can be seen as a result of UV irradiation. Polymer degradation and stability can be caused by the scission of the urethane group and photooxidation of the central CH2 group between the aromatic rings.

Table 3 below presents a summary table (compared with Blend 1 and Blend 2 presented in this work) of the mechanical properties of PP/TPU blends, including typical values for tensile strength and elongation at break, with and without compatibilizers. These values are representative of results reported in the literature for various blend ratios and compatibilizer additions [48,49,50]. All values and trends are based on peer-reviewed studies and comprehensive reviews of PP/TPU blend mechanical properties.

## 4. Conclusions

Based on the findings, exposure to UV radiation induced both physical and chemical changes in the analyzed TPU/PP blends. For instance, the SEM results revealed surface defects, with more severe damages observed in the samples of Blends 2 and 4. The FTIR analysis allowed us to identify greater chemical degradation in the samples of Blends 2 and 4.

After 320 h of exposure to UV radiation, the hardness of the samples was not significantly affected. This may indicate that the samples’ degradation process occurred superficially and did not significantly affect the bulk material. Moreover, the results of the tensile tests showed that the samples of Blends 2 and 4 experienced a noticeable reduction in maximum tensile strength after being exposed to UV radiation. The evaluated blends exhibited good performance at low deformations (50%). However, additional compositions should be assessed to confirm the effect of the PE masterbatch UV in blends at higher deformations (maximum tensile strength).

According to the thermogravimetric analysis we conducted, exposure to UV radiation did not alter the thermal stability of the material. Consequently, there were no significant changes in the chemical structure of the material that would affect its thermal behavior. Blend 2 (97TPU/3MB) demonstrated the highest tensile strength both before and after UV exposure, as shown in Figure 16. The incorporation of polyethylene (PE) as a masterbatch appears to enhance mechanical performance, likely due to its flexibility, which may help accommodate deformation mismatches that occur with other polymers. This improvement in mechanical behavior suggests that using PE-based masterbatches can contribute to the development of advanced polymeric compounds with enhanced mechanical, thermal, and barrier properties. Based on the results obtained, Blend 2 is recommended for applications that require enhanced resistance to UV radiation.

Finally, degradation was found to be more pronounced in blends 2 and 4, which had the highest percentage of TPU by weight and the lowest percentage of additives (known to inhibit UV radiation-induced degradation). The degradation was more pronounced for blends 2 and 4, which are the samples with the highest percentage by weight of TPU and the lowest percentage by weight of additives against degradation by UV radiation. This means that an adequate formulation of additives can effectively inhibit the photo-oxidative effects caused by UV radiation without affecting the physical and chemical properties of the material.

The incorporation of maleic anhydride (MA) and ultraviolet (UV) stabilizers into TPU/PP polymeric blends has demonstrated a significant impact on interfacial adhesion, thermal stability, and resistance to photodegradation [51]. The use of maleic anhydride (MA) as a compatibilizer, in combination with various UV stabilizers, presents a promising strategy for enhancing the mechanical and chemical properties of these multiphase polymer systems, particularly in industrial applications exposed to aggressive atmospheric environments. In this context, **Blend 1**, or a formulation with a higher polypropylene (PP) content, may serve as an effective alternative for incorporating MA to achieve a balanced combination of UV protection and mechanical performance. Therefore, the incorporation of MA and its detailed evaluation could be considered a valuable direction for future research stemming from this study.

## Figures and Tables

**Table 1:** Composition of the evaluated blends.

Blend	TPU (wt.%)	PE Masterbatch UV (wt.%)	PP (wt.%)
1	91	4.5	4.5
2	97	3	-
3	90	4	6
4 * 100% Material TPU	100	-	-

* Referred to in the different tests as ’Blend 4’: Blend 4 is a 100% material TPU composition; however, it is not a blend, but was denominated as a blend for different tests to compare with other blends with different PE and PP compositions.

**Table 2:** Maximum temperature of degradation of the evaluated blends before and after 320 h of exposure in a UV radiation chamber, along with their respective weight loss.

Sample	Before UV Exposure		After UV Exposure	
	T_max_ of Degradation (°C)	Weight Loss (%)	T_max_ of Degradation (°C)	Weight Loss (%)
Blend 1	405.5	41.0	405.6	41.7
Blend 2	403.3	45.3	404.7	54.7
Blend 3	401.8	40.0	403.9	41.8
Blend 4	400.8	48.8	401.5	48.6

**Table 3:** Summary table of the mechanical properties of PP/TPU blends.

Blend Composition.	Compatibilizer	Tensile Strength (Mpa)	Elongation at Break (%)	Notes
PP/TPU/70/30	None	27 *	315 *	Strength and ductility decrease with more PP
PP/TPU/80/20/EC4 (1%)	Ethylenic copolymer (EC4)	34.5 *	440 *	Compatibilizer improves both strength and elongation
PP/TPU/PP-g-NH2 70/25/5%	PP-g-NH2	21	860	Strong compatibilization effect
Blend 1: TPU/PP/PE Masterbatch UV (wt.%)/91/4.5/4.5	PE Masterbatch UV	Before UV Exposure: 22 After UV exposure: 21	Before UV Exposure: 235 After UV exposure: 205	Blend 1 is recommended for applications that require enhanced resistance to UV radiation
Blend 2: TPU/PE Masterbatch UV (wt.%)/97/3	PE Masterbatch UV	Before UV Exposure:44 After UV exposure: 29	Before UV Exposure:283 After UV exposure: 241	Blend 2 is recommended for applications that require enhanced resistance to UV radiation

* Tensile strength and elongation at break generally decrease as the proportion of PP increases in the blend without compatibilizers.

## Data Availability

Data are contained within the article.
